# Editorial

**Published:** 1873-12

**Authors:** 


### Editorial.

### ***Historical Memoranda of this Journal.***

The first number of this JOURNAL was issued in April, 1844, with Prof. Jas. V. Z. Blaney as editor, and entitled *The Illinois and Indiana Medical and Surgical Journal.* The first paragraph of Dr. Blaney's Introductory we reproduce:

"It is with pleasure, if not with pride, that we announce to the medical public the first number of the first Medical Journal that has been issued in the State of Illinois. Such an announcement may, perhaps, by some be thought premature. So indeed did it appear to us when our attention was first called to the subject. More mature reflection, however, convinced us that such an opinion was erroneous; and that it is so, must appear to all who will consider the condition of the medical profession of the West—their distance from the sources of improvement and from each other, and the necessity of disabusing public opinion of the impositions of empirics, for the spread of whose doctrines new countries afford peculiar facilities."

Those were the days when there were no railroads "out West," and the editor announced that his periodical "would be read in portions of the West almost inaccessible to the rest," and this proved true.

It started as a sixteen-page pamphlet, but of the same shape it has ever since retained. Among the prominent contributors to the first volume we notice Daniel Brainard, Wm. B. Herrick, John Evans, John McLean, Graham N. Fitch, Daniel Meeker, Austin Flint, J. H. Bird, with others whose names have since become widely known to the profession.

In 1846, the unfortunate idea of a "New Series" was first adopted, Drs. Brainard, Herrick and Evans becoming associated with Dr. Blaney as editors. The April number of 1846 was numbered Volume 1, New Series. The volume was issued in bi-monthly numbers of 96 pages each.

The same conduct and issue was continued until April, 1848, when Drs. Herrick and Evans took the editorial control alone, and the name was changed to the *Northwestern Medical and Surgical Journal.*

At the commencement of the next volume, for 1849 and 1850, Dr. Edwin G. Meek took the place of Dr. Herrick.

In May, 1851, Dr. Evans took the sole editorship.

May, 1852, Drs. W. B. Herrick and H. A. Johnson accepted the editorial tripod together.

In 1853, the volume, which had previously extended until May, was closed at December, and since that time the initial number has been issued in January.

The title page of our copy of the JOURNAL for 1854 is wanting, but in the text we find the name of Dr. N. S. Davis as editor, and from the frequent presence of the initial J. we think Dr. Johnson was still acting in the editorial capacity. This was clearly the case in 1855, numbered "Vol. IV, New Series;" so, also, in 1856, but in 1857 Dr. Johnson retired, leaving Dr. Davis sole editor.

In 1858, Dr. W. H. Byford became his associate, and the name was very properly changed to "Chicago Medical Journal," but, unfortunately, another "New Series" commenced with this as Volume I.

In 1859, Daniel Brainard again took the editorial pen, with Drs. W. Godfrey Dyas and Edwin Powell as associates.

In 1860, Drs. Dyas and Powell were replaced by Ephraim Ingals, and we find on the title page, "Vol. 3, New Series; Whole Series, Vol. 17."

In 1861, Dr. J. Adams Allen took the place of Dr. Ingals, and Dr. Brainard retained but a nominal connection.

In 1864, DeLaskie Miller, M.D., and Ephraim Ingals, M.D., assumed the control, continuing through that year and 1865.

In 1866, Drs. E. L. Holmes, R. M. Lackey and Henry M. Lyman, took joint and several possession of the quills, but did not murmur when, in 1867, the present senior editor (Dr. Allen) resumed his seat in the *sanctum*, "solitary and alone."

In 1868, the experiment of issuing in semi-monthly parts was inaugurated, and for a time promised well—indeed, that year and the first half of the next were seasons of the highest prosperity for the JOURNAL. Unhappily, a change of publishers brought "a chilling frost." *De mortuis nil*, etc. But it is due to the living to say that the temporary publisher, having secured all the accounts and books of the JOURNAL, harassed those in arrears for subscriptions and advertisements, used the editor's name everywhere most unwarrantably, ran in debt for about all the printing that was done, delayed the publication most grossly, and, to make a long story short, after defaulting to the editor several thousands of dollars and endangering the very life of the JOURNAL, left the State and ultimately hung himself in St. Louis.

But after a short vacation the present publishers were engaged, and with the brief exception of the fire months, when our mail book and everything else was destroyed, everything has moved on with all the precision of machinery, and we do not hesitate to say that the handsomest medical journal the country affords has been presented to its readers. Of its contents it need only be said: Let the reader mark, learn and inwardly digest them, and he will be profited thereby.

In 1869 the senior editor associated with himself Walter Hay, M.D., whose work is everywhere to be seen on the pages of the JOURNAL, and who now adds largely to its value by assuming the laboring oar. A.

We take great pleasure in announcing to our readers, that the paper of Dr. Bartlett, upon the relations of certain microscopic spores found in the Mississippi bottoms, to malarial fevers, which is referred to in the proceedings of the Society of Physicians and Surgeons published in the current No., will constitute the leading article in the January No. of the JOURNAL for 1874.

The author of this paper has expended many months of patient labor and careful experiment upon the subject comprised in his treatise.

To those who know Dr. B., it is unnecessary for us to guarantee the honesty and fidelity of the work which he has accomplished. To those who do not, we feel sure that the narration of his experiments will carry with it the evidences of both. The paper will be inaccessible except to readers of the JOURNAL. H.

*The Chicago Medical Examiner*, of Nov. 15, contains a very apt editorial upon the subject of "Medical Books as Advertisements for their Authors," which seems to bear internal evidences of having been written *at* a particular individual. Had we space to spare, we would take pleasure in copying it in full for the benefit of some of our older readers, who would appreciate it as it deserves. But pledged as we are to avoid personalities, we refrain. In his narration of the various forms which the "advertising dodge" assumes, the editor omits two which have come within our personal observation, *i. e.*, publishing the author's autobiography illustrated with portrait, of which we have seen nearly a quarter of a cord of unsold copies; the other is, trying to sell an unsaleable book after its condemnation by reviewers, at a reduced price. We hope the editor will continue to show up all such shams, and wish him most cordially "more point to his pencil." H.

With the current No., the JOURNAL closes the thirtieth year of its life. To us, one of the most satisfactory of all, and to our readers we believe not less so, if such conclusion may be legitimate from the substantial tokens of approval which they have tendered it. For that approval, together with the advancement of medical science, and the elevation of the standard of medical journalism, we have labored assiduously, conscious that to merit the former, we must, to some degree, accomplish the latter.

It is not alone in tokens of approval such as these that we find our greatest source of congratulation, but in the hearty spirit of co-operation with which our correspondents have lightened the editorial burthen, in contributing to the JOURNAL original articles rapidly increasing in number and value. For those accepted and published we return our thanks, and at the same time regret the necessity which has compelled us to decline others. We have now on file a number of valuable communications which will appear early in 1874, and are well worthy of careful study.

But with all these sources of congratulation and hope, we cannot, in laying down, for this year, the editorial pencil, now well-nigh worn to a stump, refrain from quoting one of the chrysostoms of English literature, knowing well that, "to innumerable readers this is a satisfying consummation; that innumerable readers consider us during these current months, but as an uneasy interruption to their ways of thought and digestion, not without a certain irritancy and even spoken invective. For which, as for other mercies, ought he not to thank the Upper Powers? To one and all of you, O irritated readers, we, with open heart and outstretched arms, will wave a kind farewell.

> "Ring out the old, ring in the new,
> * * * * * *
> The year is going, let him go;
> Ring out the false, ring in the true!"

H.

***"Brown-Sequard's and Seguin's Journal,***

> '*The Archives of Scientific and Practical Medicine,*' ceases with the Nov. (the sixth) number. Too much nerve force and too high temperature for this climate."—*Clinic, Nov.* 1.

"'Tis true, 'tis pity, and pity 'tis, 'tis true." As somebody said about facts, so much the worse for the climate. It furnishes a sad commentary upon the educational necessities of the profession, who write to medical editors, who venture to furnish only occasionally a scientific article, for "something lighter," and who utterly refuse to support one who makes his journal the medium of scientific culture alone.

We hailed the first No. of the *Archives of Scientific and Practical Medicine* with joy, as supplying a great void in American medical literature. We forwarded the subscription price at once with pleasure, and have read every number with gratification. We cannot complain of having lost any "nerve force" in its perusal, and have rather luxuriated in what the *Clinic* calls the "high temperature," as supplying a delightful change from the "heavy wet" of many of our cotemporaries.

At the funeral of the "*Archives,*" we wish to be counted in as "mourners," and yet while we mourn, it is not without hope, and we sincerely pray *ut resurget.* H.

---

***Publishers' Notice.***

In forwarding the present No. of the JOURNAL to our subscribers of the year 1873, we desire that the expression of our gratitude for their kind patronage should accompany it. At the beginning of the current year, we congratulated ourselves upon the pleasant auspices under which the new year was commenced, and the assurances which we felt enabled to make to our readers that the JOURNAL should be more than ever worthy of their patronage.

That the first have been realized we are assured by our cash balance. The fulfillment of the latter is confirmed by our rapidly increasing subscription list.

The condition and prospects of the JOURNAL have never been so prosperous and flattering as now, and we feel justified in renewing our promises of the past year with even greater assurance of our ability to fulfill them.

The JOURNAL has been brought to its present prosperous condition only by maintaining inflexibly our rule to furnish it to advance subscribers alone. We are thus enabled to return to them the full value of their subscription, without the necessity of "watering the stock."

The January No., therefore, will be sent only to those who remit the subscription price in advance.

---

### *A Bill to Promote the Science of Medicine and Surgery in the State of Illinois.*

SECTION I. It shall be lawful in cities and counties whose population exceeds twenty thousand inhabitants, for superintendents of penitentiaries, wardens of poorhouses, coroners and city undertakers, to deliver to the professors and teachers in medical colleges and schools in this State, and for professors and teachers to receive, the remains or body of any deceased person, for purposes of medical and surgical study: *provided*, that said remains shall not have been regularly interred, and shall not have been desired for interment by any relative or friend of said deceased, within twenty-four hours after death: *provided*, *also*, that the remains of no person who may be known to have relatives or friends shall be so delivered or received without the consent of said relatives or friends: *and provided*, that the remains of no one detained for debt, or as a witness, or on suspicion of crime, or of any traveler, or of any person who shall have expressed a desire in his or her last sickness that his or her body may be interred, shall be delivered or received as aforesaid, but shall be buried in the usual manner: *and provided*, *also*, that in case the remains of any person so delivered or received shall be subsequently claimed by any surviving relative or friend, they shall be given up to said relative or friend for interment.

SEC. 2. And it shall be the duty of the said professors and teachers decently to bury in some public cemetery, the remains of all bodies after they shall have answered the purposes of study as aforesaid; and for any neglect or violation of the provisions of this act, the party so neglecting shall forfeit and pay a penalty of not less than twenty-five nor more than fifty dollars, to be sued for by the health officers of said cities, or other places, for the benefit of their department.

SEC. 3. The remains or bodies of said persons as may be so received by the professors and teachers, as aforesaid, shall be used for the purposes of medical and surgical study alone, and in this State only; and whoever shall use such remains for any other purpose, or shall remove such remains beyond the limits of this State, or in any manner traffic in the same, shall be deemed guilty of a misdemeanor, and shall, on conviction, be imprisoned for a term not exceeding one year in a county jail.

SEC. 4. Every person who shall deliver up the remains of any deceased person in violation of, or contrary to, any or all of the provisions contained in the first section of this act, and every person who shall receive said remains, knowing the same to have been delivered contrary to any of the provisions of said section, shall each and every of them be deemed guilty of a misdemeanor, and shall, on conviction, be imprisoned for a term not exceeding two years in a county jail.

The above bill passed the lower house of the Illinois Legislature at its last session, and was introduced into the Senate, where those having it in charge found its defeat inevitable, owing to the absence of a number of Senators favoring the bill. It was, on motion, re-referred to the committee, and so stands for action by the adjourned session of the Legislature, which meets in January next. It passed the House by the flattering majority of 102 to 31, which gave confidence to its friends that the dark days for Anatomy had passed. The object of this communication is to appeal to every Alumnus of Rush Medical College, as well as every member of our profession in this State, to make a personal effort to secure its passage. It can be done by a united effort, brought to bear on every Senator in the State. Make this a personal matter, and *attend to it at once.* Not only use your own influence, but bring every other person's you can to bear on it, in its favor. Obtain a pledge to vote for it; or, if you cannot do that, after exhausting your best efforts, at least get them to promise *not to vote against it.*

The arguments for it need not be suggested to you, especially those who have risked their lives and liberty obtaining knowledge it were a crime against man and the State to be without. The bill is as unobjectionable as it is possible to make one of its kind, and I think, by a concentrated and determined effort made on our law-givers, we will succeed in obtaining this indispensable part of successful medical teaching, as well as rid our statute books of one of the disgraces of civilization. "Boys, help us."

R. L. REA.

